# Dopamine D2 receptor polymorphisms and susceptibility to alcohol dependence in Indian males: a preliminary study

**DOI:** 10.1186/1471-2350-11-24

**Published:** 2010-02-11

**Authors:** Pushplata Prasad, Atul Ambekar, Meera Vaswani

**Affiliations:** 1 National Drug Dependence Treatment Centre, All India Institute of Medical Sciences, New Delhi - 110029; India

## Abstract

**Background:**

Dopamine is an important neurotransmitter involved in reward mechanism in the brain and thereby influences development and relapse of alcohol dependence. The dopamine D2 receptor (*DRD2*) gene on chromosome 11 (q22-q23) has been found to be associated with increased alcohol consumption through mechanisms involving incentive salience attributions and craving in alcoholic patients. Therefore, we investigated the association of three single nucleotide polymorphisms (SNP) in *DRD2 *gene with alcohol dependence in the north Indian subjects.

**Methods:**

In a retrospective analysis, genetic association of three polymorphisms from *DRD2 *gene with alcohol dependence was investigated using a case-control approach. Alcohol dependence was determined by DSM-IV criteria and a total of 90 alcoholics and 60 healthy unrelated age-matched control subjects were recruited. Odds ratio and confidence interval was calculated to determine risk conferred by a predisposing allele/genotype/haplotype. Logistic regression analysis was carried out to correlate various clinical parameters with genotypes, and to study pair-wise interactions between SNPs.

**Results:**

The study showed a significant association of -141C Ins allele and a trend of association of TaqI A1 allele of *DRD2 *with alcohol dependence. Haplotype with the predisposing -141C Ins and TaqI A1 alleles (-141C Ins-A-A1) seems to confer ≈ 2.5 times more risk to develop alcohol dependence.

**Conclusions:**

The study provides preliminary insight into genetic risk to alcohol dependence in Indian males. Two polymorphisms namely, -141C Ins/Del and TaqI A in *DRD2 *gene may have clinical implications among Indian alcoholic subjects.

## Background

Alcohol dependence (AD) is a common but complex trait with an estimated 6.5% lifetime prevalence in the general population. Demographic data indicating higher concordance rate for monozygotic twins when compared with dizygotic twins suggest an important genetic contribution to the pathogenesis of alcohol dependence [1]. Approximately 50-60% of the population variance in alcohol dependence is accounted for by genetic factors [2], but this influence is almost certainly due to the combined effects of multiple genes, each exerting a small individual effect and interacting with other genes and environment [3].

The central dopamingeric system is widely considered to play a crucial role in development of dependence to a range of psychoactive substances including opiates, cocaine, nicotine and alcohol [4-6]. The dopaminergic system influences/regulates brain reward mechanism [7,8] and is considered a strong candidate for alcohol dependence. Alcohol, by stimulating dopamine receptors, promotes dopamine release in the ventral striatum leading to increased alcohol consumption through mechanisms involving incentive salience attributions and craving [9]. Clinical investigation involving analysis of receptor density and function has implied that dopamine D2 receptor (*DRD2*) density and function being lower among alcoholics may be responsible for craving and subsequent relapse [8]. The study of single nucleotide polymorphisms (SNPs), in dopaminergic pathway genes has shown that inheritance of different SNPs can increase or decrease the risk of alcoholism [10]. Consequently, in several populations world over, genetic association studies have been carried out to determine polymorphisms in genes encoding dopaminergic system and susceptibility to alcohol dependence [11-15]. Previous genetic association studies of the dopamine receptors (D1-D5) and transporter protein (DAT) have indicated that *DRD2 *is involved in susceptibility to alcoholism [16]. Presence of such clinical and genetic evidences has implicated *DRD2 *gene polymorphisms as strong candidates for alcoholism and therefore, they have been most widely studied.

Since 1990, many studies have addressed possible association of *DRD2 *polymorphism with alcoholism. However, findings of these studies have largely remained controversial. Inconsistent results have been explained in terms of *DRD2 *mediated "reward deficiency" syndrome, and/or population stratification bias [17]. Most of the positive findings are related to European or European American populations where as studies in Atayal natives of Taiwanese populations have generally been negative [18]. The *DRD2 *gene has three most commonly investigated polymorphisms (-141C Ins/Del, TaqI B and TaqI A) and the results are equivocal for their association with alcohol dependence [11,12]. The promoter polymorphism (-141C Ins/Del, rs1799732) of the *DRD2 *gene involving the insertion (Ins)/deletion (Del) of a cytosine is related to receptor density [19]. SNP TaqI B being closer to the regulatory and structural coding regions (5' region) of the gene [20] is considered to play important role in transcription regulation. TaqI A SNP is regarded as the most dramatic polymorphism of *DRD2 *for its highly inconsistent association with AD. The A1 allele of TaqI A SNP has been found to be associated with low D2 dopamine receptor availability in the striatum [21]. Recently, it has become evident that the TaqI A polymorphism previously considered to be present in 3' UTR of *DRD2 *is actually located in a nearby novel gene (in reverse orientation) named ankyrin repeat and kinase domain containing (*ANKK1*) where it causes a missense substitution [22]. TaqI A SNP of the *ANKK1 *gene is associated with increased striatal activity of aromatic L-amino acid decarboxylase, the final enzyme in the biosynthesis of dopamine [22].

India, representing 1/6^th ^of the world population, has sudden surge in alcohol dependence and related problems. As per the estimates of the National Survey, about 21% of male adult population could be described as 'Current User' (defined as consumption at least once in preceding one month), which translates into about 62 million people, out of which about 17% are dependent users [23]. However, only one genetic association study to unravel genetic predisposition in Indian alcoholic population has been carried out to date [24]. In view of the paucity of Indian data on genetic polymorphism of alcohol dependence, we aimed to investigate the association of *DRD2 *gene polymorphisms in alcohol dependent subjects of north Indian origin.

## Methods

### Study population

Ethical committee clearance from the All India Institute of Medical Sciences (AIIMS) was obtained before initiation of the study. A written informed consent was obtained prior to recruitment of case and control subjects. The process of clinical assessment was carried out by qualified psychiatrist. One hundred and forty male alcohol dependent subjects attending the out patient department (OPD) at National Drug Dependence Treatment Centre, All India Institute of Medical Sciences (AIIMS) were screened. Out of these, 37 were polysubstance users and 13 had co-morbid depression/anxiety/or schizophrenia, and therefore, were excluded from the study. Remaining 90 unrelated outpatients with alcohol dependence, in the age range of 18-60 years, were enrolled as cases (AD). A total of 60 unrelated healthy male employees of the hospital, without any history of substance use (except nicotine) were included as controls (C). Since nicotine use is widely prevalent among males in India, neither case nor controls were excluded on the basis of their nicotine use. All individuals regarded themselves to be of north Indian origin. Diagnosis of alcohol dependence was determined by experienced psychiatrists using the DSM-IV criteria (American Psychiatric Association, 1994; [25]). The study was conducted in accordance with worldwide good-clinical-practice (GCP) standards and confirmed to acceptable ethical standards as outlined by local requirements and the Declaration of Helsinki (World Medical Association, 1989).

All patients were assessed for alcohol use parameters using AUDIT [26], and a semi-structured questionnaire. The semi-structured questionnaire included items on clinical details like ethnicity, family history, age at first use of alcohol, quantity of alcohol consumption (g/day), duration of alcohol use, duration of alcohol dependence, age at onset of dependence, presence/absence of delirium and any other psychiatric or physical illness. The same semi-structured questionnaire was used for assessment of the control population as well. The assessments also included liver function tests (LFT) such as serum proteins, albumin, bilirubin, glutamic oxaloacetic transaminase (SGOT) and glutamic pyruvic transaminase (SGPT), gamma-glutamyltransferase (GGT), and mean corpuscular volume (MCV). LFT were estimated on autoanalyser using bio-chemical kits from Boehringer Mannheim kits (Germany).

### Genetic Analysis

DNA was isolated from the lymphocytes using the conventional phenol-chloroform organic extraction method [27] and used for genetic analysis. Three SNPs were selected from the *DRD2 *locus based on prior published association reports, information content, minor allele frequency (MAF), linkage disequilibrium (LD) structure, and validation evidence. SNP genotyping was done using polymerase chain reaction (PCR)-restriction fragment length polymorphisms (RFLP) approach following previously published method [28,29]. The digested PCR products were resolved on 2-3% agarose gel stained with ethidium bromide. Genotypic profiles obtained for each of the polymorphisms are presented in Table 1.

**Table 1:** Genotypic profiles obtained for *DRD2 *polymorphisms

SNP	Primers	Annealing temp./R.E./fragment size
-141C Ins/Del	F:5'GACCCAGCCTGCAATCAC3' R:5'AGGAGCTGTACCTCCTCGG3'	57°C/Bst NI Ins C = 124, 32 Del C = 156

TaqI B	F:5'GATGTGTAGGAATTAGCCAGG3' R:5'GATACCCAGTTCAGGAAGTC3'	56°C/TaqI G = 459 A = 267,192

TaqI A	F:5'CCGTCGACGGCTGGCCAAGTTGTCCA 3' R:5'CCGTCGACCCTTCCTGAGTGTCATCA3'	58°C/TaqI T (A1) = 310 C (A2) = 180,130

### Position of the three SNPs studied in DRD2 along with their identification number is indicated below

**-**141C Ins/Del, promoter (**rs1799732**); G>A, TaqI B, 1 kb upstream from exon 2 (**rs17294542**); and T>C, TaqI A, 10 kb downstream from exon 8 (**rs1800497**).

### Statistical Analysis

Comparison of all clinical variables between alcohol dependent (AD) and control (C) subjects was carried out by χ^2 ^test for nominal variables or student's t-test for continuous variables. Continuous variables with skewed distribution were compared by Mann Whitney's U test. Hardy-Weinberg equilibrium (HWE) was tested for each of the genetic marker. Allelic and genotypic associations of SNPs were evaluated by Pearson's χ^2 ^test followed by odds ratio and 95% CI computation. Fisher' exact test was applied for SNPs having cell value less than 5 for any one (out of three) genotypes. Power of the sample size for each of the SNPs was calculated using PAWE software version 1.2 [30,31]. Haplotype analysis was performed using PHASE-standard analysis version 2.0.2 [32,33]. Chi-square values were derived from a series of 2 × 2 contingency tables based on the frequency of each haplotype versus all others between the AD and the control groups. Multiple logistic regression (MLR) analysis were carried out to correlate various clinical parameters with genotypes and to study pair-wise interactions between SNPs. P values < 0.05 were considered significant.

## Results

### Clinical analysis

A comparison of demographic and clinical characteristics between cases and controls are presented in Table 2. As can be seen in the table, on almost all demographic parameters, the control group was largely similar to the AD group. However, much larger proportion of AD subjects had education up to primary school (i.e. five years of formal schooling) only. The values for various clinical parameters such as AUDIT score, alcohol intake (g/day) SGOT, SGPT, GGT, cholesterol, and triglycerides (TG)] were significantly higher (P < 0.01) among AD (cases) as compared to the control group.

**Table 2:** Demographic and Clinical characteristics of the study population presented as Mean ± SD

Characteristics	AD (Case)	Controls
Age (y)	36.75 ± 9.27	35.17 ± 11.59

Married	86%	80%

Employed	96%	100%

Education up to Elementary School Only	37%	1%

Living with family	85%	82%

Age at first use of alcohol	19.89 ± 5.56	24.30 ± 6.28

Audit Score	32.12 ± 5.59	1.04 ± 1.58

Alcohol intake (g/day)	183.89 ± 104.54	2 ± 0.5

T.Bil. (mg/dl)	1.96 ± 0.78	0.7 ± 0.2

T.Prot. (g/dl)	7.02 ± 1.04	8.00 ± 0.76

Alb. (g/dl)	4.19 ± 0.54	4.52 ± 0.30

SGOT (U/l)	84.21 ± 27.76	27.02 ± 09.71

SGPT (U/l)	83.13 ± 26.29	28.30 ± 11.49

Chol. (mg/dl)	181.49 ± 53.11	132.62 ± 12.01

GGT (U/l)	210.02 ± 42.02	24.95 ± 11.88

TG (mg/dl)	198.58 ± 108.01	121.59 ± 11.80

### Genetic analysis

All the three SNPs analyzed in the study were in HWE in the base line control population. No significant pair-wise linkage disequilibrium was observed between the three polymorphisms. Allele and genotype frequencies of SNPs in *DRD2 *and their association status with AD are presented in Table 3.

**Table 3:** Allele and genotype frequencies of SNPs in *DRD2 *and their association status with AD

SNPs	Genotypes			Statistics	Alleles		Statistics	Power (*G %)
**-141C Ins/Del**	**Ins/Ins**	**Ins/Del**	**Del/Del**		**Ins**	**Del**		

**AD**	0.61	0.32	0.07	**χ^2 ^= 11.0, P < 0.01**	0.77	0.23	**χ^2 ^= 4.3, P < 0.05**	62
				
**Controls**	0.57	0.16	0.27		0.65	0.35		

**TaqI B**	**GG**	**GA**	**AA**		**G**	**A**		

**AD**	0.07	0.37	0.56	χ^2 ^= 0.11, P = 0.94	0.25	0.75	χ^2 ^= 0.12, P = 0.72	6
				
**Controls**	0.11	0.31	0.58		0.27	0.73		

**TaqI A**	**A1A1**	**A1A2**	**A2A2**		**A1**	**A2**		

**AD**	0.04	0.45	0.51	**Fisher's exact test P < 0.05**	0.27	0.73	**Fisher's exact test p = 0.08**	16
				
**Controls**	0.07	0.30	0.63		0.22	0.78		

*G: the power of SNP to detect association, calculated using PAWE software

Of the three polymorphisms analyzed in *DRD2 *gene, significant allelic and genotypic association of -141C Ins/Del SNP was observed with alcohol dependence. SNP TaqI B did not show any allelic or genotypic association. For TaqI A SNP, genotype Taq A1A1 was present in negligible frequency. Categorical analysis showed significant association of Taq A1/A2 genotype (Fisher's exact p < 0.05) with alcohol dependence. In addition, a trend towards association of allele TaqI A1 with AD was also found (Table 3).

Significantly associated haplotypes of *DRD2 *gene present in AD subjects are shown in Table 4. Two (-141C Ins-A-A1, and -141C Del-A-A2) out of eight possible haplotypes in these cases were significantly associated with AD, former being predisposing and latter protective to AD (Table 4).

**Table 4:** Significantly associated SNP haplotypes in *DRD2 *gene

^a^**Haplotype**	C (120)	AD (180)	χ^2^	P	O.R (95% CI)
-141C Ins-A-A1	13(0.11)	38 (0.21)	5.39	0.02	2.20 (1.19-4.33)

-141C Del-A-A2	12 (0.10)	06 (0.03)	5.67	0.01^b^	0.31 (0.11-0.85)

^a^Order of SNPs in the *DRD2 *haplotypes: -141C Ins/Del - G>A (Intron 1) - TaqI A (Intron 7)

^b^Significant after Bonferroni correction α = 0.01

A combined analysis involving genetic and crucial clinical/laboratory parameters (SGOT, SGPT, GGT, and alcohol consumed/day) indicated that the sub-group of AD patients with -141C Ins/Ins genotype (of -141C Ins/Del polymorphism) had significantly higher values (P < 0.01) of SGOT, SGPT, and GGT as compared to those with -141C Ins/Del and -141C Del/Del genotypes.

To assess synergistic effect of various clinical and genotypic parameters (analyzed in this study), multiple logistic regression analysis (MLR) was carried out. Disease status was taken as the dependent parameter, and genotypes, alleles (-141C Ins/Del, TaqI B, TaqI A) and clinical parameters were taken as the independent variables. A significant association of SNP -141C Ins/Del of *DRD2 *gene (P < 0.05; OR 0.19, 95%CI 0.06-0.6) was seen with AD. Further categorical analysis identified -141C Ins allele (P < 0.05; OR 1.81, 95%CI 1.03-3.19) to be predisposing to AD. No significant interaction between any of the polymorphisms/genes was observed when pair-wise interactions between different polymorphisms were carried out by multiple logistic regression analysis.

## Discussion

In relation to alcohol dependence, the research provides unambiguous evidence that genes and their polymorphisms play an important role in its development [34]. Alcohol increases synaptic dopamine, which reinforces self administration. In the ventral striatum and particularly in the nucleus accumbens, different drugs of abuse stimulate dopamine release thereby reinforcing drug consumption. The down regulation of dopamine D2 receptors in these areas of the brain have been associated with alcohol craving and an increase in the processing of alcohol-related stimuli in the medial prefrontal cortex [9]. Dopamine D2 receptor gene (*DRD2*) encodes a G protein-coupled receptor located on post-synaptic dopaminergic neurons, which plays a central role in reward-mediating mesocorticolimbic pathways [20]. Possible association between polymorphisms at the *DRD2 *gene and alcohol dependence has been extensively investigated since first proposed in 1990 [35].

The present study tested association of three polymorphisms -141C Ins/Del, TaqI B and TaqI A, present at *DRD2 *gene locus with alcohol dependence in north Indian subjects.

To the best of our knowledge, this study constitutes the first association report with regard to the first two SNPs (-141C Ins/Del, TaqI B in DRD2 gene) with alcohol dependence from India. Although, association of TaqI A with AD has not been carried out in the north Indian population by anyone to date, a study from south India reported no association between TaqI A and alcoholism [24].

The -141C Ins/Del polymorphism has been investigated for its association with alcoholism in several studies across different populations [1,36,37], however, the results are inconsistent. This promoter polymorphism plays an important role in D2 receptor expression. An *in vitro *analysis suggested that -141C Ins/Del polymorphism of *DRD2 *alters its transcriptional activity and thus regulates the expression of *DRD2 *receptor [19]. An imaging study carried out in healthy volunteers demonstrated increased striatal receptor density in -141C Del carriers [38]. In a genetic association study, Samochowiec *et al*. (2000) suggested a possible role of -141C Ins/Del SNP (in particular haplotypic combination, Ins/G/A2) in German alcoholics [39]. However, in a family-based case-control study of a Polish population, they failed to replicate this finding [40]. Johann *et al*. (2005) studied the association of a -141C deletion variant (-141delC) of the DRD2 gene in well-characterized, primary chronic alcoholics of German descent and found an excess of the -141delC alleles in alcoholics with a paternal and grand-paternal history of alcoholism. They concluded that though the -141delC variant of DRD2 might be a protective factor against the development of withdrawal symptoms, it might also be a risk factor in a highly burdened subgroup of alcoholics with a paternal and grand-paternal history of alcoholism [41]. Significant association of SNP -141C Ins/Del (P < 0.05; OR 0.19, 95%, CI 0.06-0.6) in *DRD2 *gene with alcohol dependence followed by categorical analysis suggesting -141C Ins allele to be predisposing to alcohol dependence in the present study is in conformity with findings of Konishi *et al*., which indicated that -141C Ins allele is a genetic risk factor for alcoholism in Mexican-Americans [36]. Thus a significant over representation of -141C Ins allele in alcoholic subjects in our study may be correlated with decreased *DRD2 *receptor density in AD patients, which in turn stimulates craving - reward pathway - thereby promoting alcohol dependence. However, this statement should be interpreted with caution since Hirovonen *et al*., (2009) suggested that the -141C Ins/Del genotype did not influence extrastriatal DRD2 binding potential using 3D-PET imaging in healthy male controls [42]. Further, our results also suggest that presence of -141C Del/Del genotype confers protection from AD and -141C Ins allele carriers are at higher risk for developing AD as compared to -141C Del carriers. As the power to of the sample size to detect association of this SNP was moderate (G = 62%), the observed genotypic and allelic association with AD needs to be interpreted cautiously.

SNP TaqI B is closer to the regulatory and structural coding regions (5' region) of the *DRD2 *and thus supposed to play an important role in gene function [27]. It has been rarely investigated for its association with AD. Two studies [36,43] carried out in Mexican-American population reported conflicting results with regard to association of this polymorphism with AD. Our observation of no allelic or genotypic association of TaqI B polymorphism with AD in north Indians concurs with Konshi *et al*. [36] reporting no positive association of TaqI B with AD in Mexican-Americans. However in a subsequent study, the same group reported an association of TaqI B polymorphism with early age of onset for alcohol drinking in Mexican-Americans [43].

*ANKK1 *rs1800497, previously called "*DRD2 *TaqI A", is one of the most frequently studied polymorphisms in *DRD2 *genetic studies. Neville *et al*. [44] determined that the *DRD2 *TaqI A is actually located not within *DRD2*, but rather within a protein-coding region, exon 8, of the adjacent *ANKK1 *gene. Blum *et al*. [35] reported the presence of A1 allele of DRD2 receptor gene classifying 77% of alcoholics, demonstrating an association of A1 allele with alcoholism. Association studies of the *DRD2 *TaqI A1 allele and alcohol consumption have remained ambiguous and controversial due to conflicting results [45]. Although the *DRD2 *TaqI A polymorphism and alcohol dependence have been studied extensively, it emerged that this polymorphism may affect substrate-binding specificity. It remains to be elucidated whether the associations with TaqI A are due to its own functionality or linkage disequilibrium with another *DRD2 *variation or with the *ANKK1 *gene [45]. Pohjalainen *et al*. [21] reported an association between A1 allele and low D2 receptor availability in healthy subjects indicating that A1 allele of the TaqI A polymorphism might be in linkage disequilibrium with a mutation in the promoter/regulatory gene that affects dopamine D2 receptor expression. This was further correlated with risk conferring nature of the A1 allele in subsequent association studies in different populations like Caucasians, Han Chinese, and Europeans [24]. However, few studies from East Asian, European and Caucasian populations reported negative association of TaqI A1 allele with alcohol dependence [46]. Recently, Samochowiec *et al*. (2006) also did not find any association of TaqI A1 with AD in a Polish population [40]. However, they observed a statistically significant preferential A2 allele transmission in DRD2 TaqI A gene polymorphism in subgroups of patients with early onset and withdrawal complications. Their results also suggested various subtypes of alcohol dependence, which differ depending on their genetic background and may confound association findings [40].

Further, Laruelle *et al*. (1998) did not find association between A1 allele and lower D2 receptor expression and suggested that D2 receptors binding potential is not affected by TaqI A polymorphism at the D2 receptor gene [47]. Our observation of association of TaqI A is due to significant excess of heterozygote Taq A1/A2 genotype in the AD population from north India which could be attributed to yet unknown selection pressure. However, a marginal association of allele TaqI A1 is in concurrence with the meta-analysis carried out by Munafo *et al*. where association of the *DRD2 *TaqI A polymorphism with alcoholism suggest that A1 allele confers modest increase in risk [48]. Considering this background, the association of TaqI A1 allele with AD in the present study could be due to the altered D2 receptor expression and reward mechanism in alcohol dependent subjects. Since the power of the sample size to detect association of this SNP was low (G = 16%), the observed association (of this SNP with AD) should not be over-interpreted.

Our observation of A2/A2 genotype being most common in North Indian population is in concurrence with Caucasian population where an excess of A2 homozygotes has been reported [49]. The A1 allele frequency ranges between 0.06 and 0.18 in populations with European ancestry, while a significantly higher frequency (0.7) is seen in native South American populations [24]. In the present study, allele frequency (0.22) observed for A1 allele is closer to European population whereas the allele frequency observed in the south Indian population (0.42) is similar to reports from south East Asian populations with allele frequency of 0.45 [24]. The observed difference in allele frequencies between north Indian and south Indian population is due, in part, to the many different waves of immigrants that have influenced the genetic structure of India. The Indo-European (IE) and Dravidian (DR) groups have been the major contributors to the development of Indian culture and society. People from southern part of India have Dravidian background, and those form northern part have Indo-European (Caucasoid) background [50].

Results of independent association of SNPs were replicated in haplotypic association. As expected haplotype containing -141C Ins and TaqI A1 alleles was found to be predisposing to alcoholism and conferred ≈ 2.5 times risk to patients with this haplotypic combination (-141C Ins-A-A1). On the other hand haplotypic combination with -141C Del and Taq A2 alleles (-141C Del-A-A2) seems to confer protection against alcoholism. Thus, our result of haplotypic association (-141C Ins-A-A1) is in partial concurrence with German population where haplotype Ins/A2 causes predisposition to severe alcoholism [36]. Inconsistency between our and the previous report [36] could be attributed to population specific differences in genetic structure.

SGOT, SGPT, and GGT are markers of alcohol related liver dysfunction. Heavy drinking causes acetaldehyde toxicity leading to liver damage. A sub-analysis based on genotypic constitution with regard to -141C Ins/Del SNP revealed significant alteration in liver functions (as indicated by higher SGOT, SGPT and GGT values) among patients with -141C Ins/Ins genotype. We propose that this finding of association of -141C Ins/Del SNP with liver dysfunction among alcoholics should be explored further, though in this study it may be a purely chance finding.

## Conclusions

Two polymorphisms namely, -141C Ins/Del and TaqI A in *DRD2 *seem to have clinical implications in the development of alcoholism. However, our results have to be viewed in the perspective of potential limitation posed by small sample size and it warrants replication in larger sample sets.

## Competing interests

The authors declare that they have no competing interests.

## Authors' contributions

All authors have read and approved the final Ms. PP was involved in the study design, carried out molecular genetics and statistical analyses, compiled the data, wrote the Ms.; AA was the clinical investigator involved in study design, defining exclusion and inclusion criteria of study subjects and was mainly responsible for identification of study subjects; MV was the principal scientist and coordinator of the project, involved in conceptualization of the project, study design, oversee complete genetic analyses in the laboratory, critical inputs and finalization of the manuscript. All authors read and approved the final paper.

## Pre-publication history

The pre-publication history for this paper can be accessed here:

http://www.biomedcentral.com/1471-2350/11/24/prepub
